# Cerebral Vasospasm Detection and Delayed Cerebral Ischemia Prediction after Aneurysmal Subarachnoid Hemorrhage: A Scoping Review

**DOI:** 10.1007/s12028-026-02509-z

**Published:** 2026-04-20

**Authors:** João Brainer Clares de Andrade, Nayara de Lima Froio, Paula Sanchez Nascimento, Sophia Oliveira Querobin, Nathalia Souza de Oliveira, Thiago S. Carneiro

**Affiliations:** 1 Departament of Neurology, Universidade Federal de São Paulo, Rua Botucatu 720, São Paulo, Brazil; 2 Department of Health Informatics, Universidade Federal de São Paulo, São Paulo, Brazil; 3 Hospital Israelita Albert Einstein, São Paulo, Brazil; 4 School of Medicine, Centro Universitario São Camilo, São Paulo, Brazil; 5 Department of Neurocritical Care, University of Florida, Gainesville, USA

**Keywords:** Subarachnoid hemorrhage, Cerebral vasospasm, Delayed cerebral ischemia, Transcranial Doppler, CT perfusion, Machine learning, Biomarkers

## Abstract

**Background/Objective:**

Aneurysmal subarachnoid hemorrhage (aSAH) is complicated by angiographic cerebral vasospasm and delayed cerebral ischemia (DCI), distinct entities with different reference standards and surveillance targets. We mapped the evidence on (1) diagnostic accuracy of vasospasm monitoring tools using digital subtraction angiography (DSA) as the reference standard and (2) detection and prediction of DCI using the 2010 consensus definition.

**Methods:**

We performed a Preferred Reporting Items for Systematic Reviews and Meta-Analyses extension for Scoping Reviews (PRISMA-ScR)-guided scoping review of PubMed/MEDLINE, Google Scholar, and PubMed Central (1 January 2005–31 January 2025). Vasospasm studies required DSA as comparator; DCI studies were categorized by outcome definition (2010 consensus vs. alternative definitions). Methodological quality was assessed with QUADAS-2 (diagnostic accuracy) and Prediction Model Risk of Bias Assessment Tool (PROBAST; prediction models).

**Results:**

From 762 records, 136 studies met inclusion criteria. For vasospasm detection (DSA reference), transcranial Doppler (18 studies; *n* = 3256) demonstrated sensitivity 66% (60–72) and specificity 97% (95–99), while computed tomography angiography (CTA) (15 studies; *n* = 2145) showed sensitivity 76% (72–80) and specificity 93% (91–95). For DCI detection (2010 consensus), CT perfusion (30 studies; *n* = 1786) yielded sensitivity 86% (78–86) and specificity 80% (75–85) with substantial protocol heterogeneity; continuous electroencephalography (cEEG) (8 studies; *n* = 523) achieved sensitivity 88% (80–94) and specificity 89% (83–93). Across 84 DCI predictor studies, machine-learning models (23 models) outperformed traditional risk factors (area under the curve [AUC] 0.68–0.88 vs. ~0.63), but only 44% underwent independent validation, and 67% were at high risk of bias.

**Conclusions:**

The evidence supports a two-track framework: DSA-referenced tools for vasospasm detection and consensus-defined markers for DCI detection/prediction. Multimodal strategies integrating physiologic and imaging data are most promising, whereas machine-learning approaches require prospective, externally validated evaluations before routine clinical deployment.

**Supplementary Information:**

The online version contains supplementary material available at https://doi.org/10.1007/s12028-026-02509-z.

## Introduction

Aneurysmal subarachnoid hemorrhage (SAH) affects approximately 9 per 100,000 person-years globally and carries a 30-day mortality rate of 35% [1]. The leading cause of morbidity and mortality is the initial severity of the hemorrhage, but secondary brain injury from cerebral vasospasm and delayed cerebral ischemia (DCI) can contribute to a considerable worsening of prognosis [2].

It is essential to distinguish between vasospasm and DCI, as these terms are often conflated but represent distinct phenomena. Vasospasm refers to angiographically documented narrowing of cerebral arteries, with digital subtraction angiography (DSA) serving as the gold standard for detection. DCI, as defined by the 2010 international consensus criteria, encompasses either: (1) cerebral infarction identified on CT or MRI not attributable to other causes, or (2) clinical deterioration (new focal neurological deficit or ≥2-point decrease in Glasgow Coma Scale [GCS]) lasting >1 hour after exclusion of other causes [3, 4]. Importantly, not all vasospasm leads to DCI, and DCI can occur without detectable vasospasm due to other mechanisms including microthrombosis, cortical spreading depolarization, and neuroinflammation [5].

Various monitoring modalities have emerged, each with unique strengths and limitations.

Vasospasm typically peaks between days 4-10 post-SAH and occurs in up to 70% of patients [6]. However, only 20-30% develop clinical DCI [7]. Early detection of vasospasm is important regardless of symptoms because: (a) asymptomatic vasospasm may progress to symptomatic disease; (b) early identification enables prophylactic intervention; and (c) vasospasm severity may inform treatment decisions [8].

The 2023 American Heart Association/American Stroke Association guidelines emphasize early detection of vasospasm as crucial for guiding therapeutic interventions, including hemodynamic augmentation, intra-arterial vasodilators, or angioplasty [5]. However, the 2010 consensus definition of DCI, while improving research standardization, relies primarily on clinical examination and CT imaging, which may miss DCI in poor-grade patients who cannot be reliably examined. Additional diagnostic modalities with variable accuracy—including TCD, CTP, EEG, and invasive monitors—are increasingly used to complement clinical assessment and may help refine DCI detection [10, 11].

Our scoping review aims to map the current evidence (2005-2025) on: (1) diagnostic accuracy of vasospasm surveillance tools, with DSA as the gold standard reference; and (2) predictors and detection methods for DCI, distinguishing studies using the 2010 consensus definition from those using other definitions.

## Methods

### Study Design

We conducted a scoping review following the Preferred Reporting Items for Systematic Reviews and Meta-Analyses extension for Scoping Reviews (PRISMA-ScR) guidelines [12].

### Search Strategy

We performed a comprehensive search across three databases from January 1, 2005 to January 31, 2025. The PubMed/MEDLINE database was searched using combinations of terms including "subarachnoid hemorrhage" OR "SAH" AND "cerebral vasospasm" OR "delayed cerebral ischemia" OR "DCI" AND various modality-specific terms.

### Eligibility Criteria

Studies were included if they met the following criteria. For vasospasm detection studies, we required the use of DSA as the reference standard. For DCI studies, we classified studies separately based on whether they employed the 2010 consensus definition or other definitions such as "symptomatic vasospasm" or clinical deterioration without imaging correlation. We included original research articles, systematic reviews, and meta-analyses published between January 2005 and January 2025 in the English language, involving adult populations (≥18 years). Seminal pre-2005 systematic reviews that remain foundational references in the field (e.g., Lysakowski et al., 2001, for TCD diagnostic accuracy) were cited for historical context but were not part of the formally included evidence set.

Studies were excluded if they involved non-aneurysmal SAH (traumatic or perimesencephalic), consisted of case reports or case series with fewer than 10 patients, were conference abstracts without full text available, or comprised editorials, commentaries, or opinion pieces. We also excluded studies focusing on pediatric populations and animal or in vitro studies.

### Study Selection

Two independent reviewers (AB and CD) screened titles and abstracts, followed by full-text review of potentially eligible articles.

### Data Extraction

A standardized data extraction form was developed and piloted on ten randomly selected studies before full implementation. Studies were classified by their outcome definition: angiographic vasospasm (DSA reference), DCI per 2010 consensus criteria, or other DCI definitions.

### Data Synthesis

Given the heterogeneity of included studies, we conducted a narrative synthesis rather than meta-analysis.

## Results

### Search Results

**Fig. 1:**
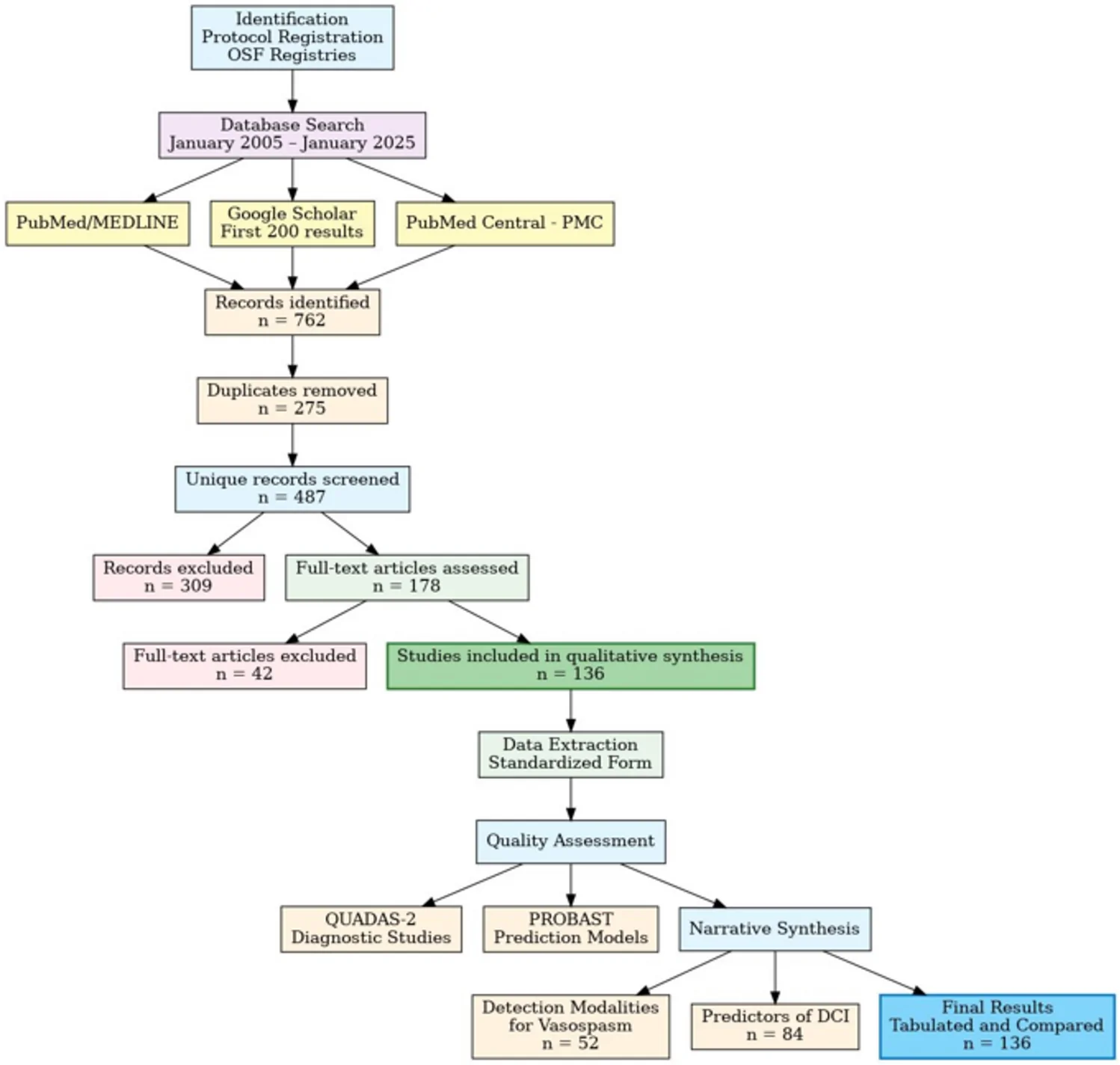
PRISMA flow diagram. Legend. Flow diagram illustrating the systematic study selection process according to PRISMA-ScR (Preferred Reporting Items for Systematic Reviews and Meta-Analyses extension for Scoping Reviews) guidelines. The search was conducted across three databases (PubMed/MEDLINE, Google Scholar, and PubMed Central) from January 1, 2005 to January 31, 2025. Initial search yielded 762 citations, with 275 duplicates removed. Title and abstract screening of 487 unique articles led to full-text review of 178 articles, with 136 studies ultimately meeting all inclusion criteria for the final analysis. Reasons for exclusion at the full-text review stage included: non-aneurysmal SAH etiology, insufficient sample size (< 10 patients), lack of diagnostic accuracy data, and conference abstracts without full text availability

The initial search yielded 762 citations. After removing duplicates (n=275), 487 unique articles underwent title/abstract screening. Full-text review of 178 articles resulted in 136 studies meeting inclusion criteria (Figure 1).

The following results are organized into two main sections: (1) Vasospasm Detection, presenting modalities evaluated against DSA as the reference standard; and (2) DCI Detection and Prediction, presenting modalities and predictors for delayed cerebral ischemia as defined by the 2010 consensus criteria.

1. Vasospasm Detection

We identified 52 studies evaluating diagnostic modalities for vasospasm surveillance. For all vasospasm detection studies, digital subtraction angiography (DSA) was used as the reference standard. DSA provides definitive diagnosis of angiographic vasospasm with 100% sensitivity and specificity by definition [15, 24]; however, its invasive nature (procedural complication rate 1.8%) [16, 18] and resource requirements limit its role as a screening tool. DSA is therefore not evaluated as a detection modality in this review but rather serves as the comparator against which all other modalities are assessed. Table 1 summarizes performance characteristics of major modalities.

**Table 1:** Diagnostic performance of vasospasm detection modalities and DCI

Modality	Studies	Patients (n)	Sensitivity (95% CI)	Specificity (95% CI)	Cost*	Major limitations
Table 1A. Vasospasm Detection (Reference Standard: DSA)						
TCD	18	3,256	66% (60–72%)	97% (95–99%)	$	Operator dependent; limited temporal windows (10–20%); proximal vessels only
CTA	15	2,145	76% (72–80%)	93% (91–95%)	$$	Radiation; contrast; misses distal spasm
Table 1B. DCI Detection (Reference: 2010 Consensus Criteria)						
CTP	30	1,786	86% (78–86%)	80% (75–85%)	$$$	Radiation/contrast; limited coverage; measures hypoperfusion not vasospasm
cEEG	8	523	88% (80–94%)	89% (83–93%)	$$$	Indirect measure; continuous monitoring; expertise required
NIRS	3	156	94% (87–98%)	86% (78–92%)	$$	Limited validation; regional monitoring only
Microdialysis	5	287	89% (82–94%)	91% (85–95%)	$$$$	Invasive; requires expertise; limited brain coverage

Table 1A: Vasospasm detection modalities evaluated against DSA as the reference standard. Table 1B: DCI detection modalities evaluated against the 2010 consensus criteria. DSA is not included as a detection modality—it serves solely as the reference standard (comparator) for vasospasm studies. CTP is presented exclusively in Table 1B as it measures hypoperfusion (a DCI marker) rather than angiographic vasospasm

*cEEG* continuous Electroencephalography, *CI* confidence interval, *CTA* computed tomography angiography, *CTP* computed tomography perfusion, *NIRS* near-infrared spectroscopy, *TCD* transcranial doppler

*Cost categories: $ =  < $500; $$ = $500–2,000; $$$ = $2,000–5,000 USD per examination

### Transcranial Doppler (TCD)

Eighteen studies evaluated TCD performance. Mean sensitivity was 66% (95% CI: 60-72%) and specificity 97% (95% CI: 95-99%) for detecting angiographic vasospasm [15, 17, 20].

### CT Angiography (CTA)

Fifteen studies assessed CTA accuracy. The 2023 systematic review by Bergin et al. (7 studies, n=856 patients) reported pooled sensitivity of 76% (95% CI: 72-80%) and specificity of 93% (95% CI: 91-95%) for moderate-to-severe vasospasm [19, 23].

## DCI Detection and Prediction

This section presents modalities for DCI detection and risk prediction, distinct from the vasospasm detection modalities above. DCI, as defined by the 2010 consensus criteria, requires evidence of cerebral infarction on imaging or clinical deterioration (new focal deficit or GCS decrease ≥2) lasting >1 hour, not attributable to other causes [4]. Studies are classified by whether they employed the 2010 consensus definition or other definitions.

### CT Perfusion (CTP)

CTP measures cerebral hypoperfusion rather than vasospasm directly, and is therefore evaluated as a DCI detection tool. The 2024 meta-analysis by Mitchelle et al. (30 studies, 1,786 patients) reported pooled sensitivity of 85.6% and specificity of 79.6% for DCI detection [21]. However, substantial heterogeneity was observed across studies (I [2] >70%), reflecting differences in CTP protocols, perfusion thresholds, and timing of assessment relative to symptom onset (Table 2).

**Table 2:** Machine learning models for DCI prediction

Study	Year	n	Input variables	Algorithm	Validation	AUC (95% CI)	Key findings
Zarrin et al. [9]	2024	846	172 ICU variables	LightGBM	External (UCLA → Vanderbilt)	0.88 (0.84–0.92)	First true external validation; ruled out verapamil need 10 d early
Hu et al. [37]	2022	404	Clinical + imaging	Random Forest	5 centers	0.86 (0.82–0.90)	20.8% higher accuracy than logistic regression
Megjhani et al. [38]	2018	488	Hourly vital signs	CNN + dictionary learning	External	0.78 (0.73–0.83)	High-frequency physiologic patterns improved prediction
Ramos et al. [39]	2019	317	Clinical + CT imaging	Random Forest	tenfold CV	0.74 (0.69–0.79)	Image features improved prediction
Tanioka et al. [40]	2019	95	Clinical + plasma proteins	Random Forest	Bootstrap	0.81 (0.72–0.89)	Biomarkers improved accuracy

Summary of machine learning models for predicting delayed cerebral ischemia after aneurysmal subarachnoid hemorrhage that have undergone validation. Models are presented in chronological order by publication year, with emphasis on those achieving external validation across multiple centers

*AUC* area under the curve (ROC curve), *CI* confidence interval; *CNN* convolutional neural network; *CT* computed tomography; *CV* cross-validation; *DCI* delayed cerebral ischemia; *ICU* intensive care unit, *n* number (sample size); *ROC* receiver operating characteristic, *LightGBM* light gradient boosting machine, *UCLA* University of California Los Angeles

It is important to emphasize that CTP does not accurately determine whether perfusion is adequate for the brain's metabolic demand—PET scanning with oxygen extraction fraction measurement remains the gold standard for metabolic assessment. CTP-derived parameters commonly used for identifying hypoperfusion include: time-to-peak (TTP) delay >4 seconds, mean transit time (MTT) prolongation, and Tmax >6 seconds [22].

A descriptive analysis by publication era revealed improving CTP performance over time: studies published before 2015 (n=12) reported pooled sensitivity of 78% and specificity of 72%, while studies published 2015-2025 (n=18) demonstrated sensitivity of 89% and specificity of 84%, likely reflecting advances in scanner technology, post-processing software, and protocol standardization.

### Continuous EEG (cEEG)

Eight studies investigated continuous EEG (cEEG) for DCI detection and prediction. Several distinct EEG patterns have been identified as markers of impending ischemia. The most consistently reported findings include: (a) diffuse or focal slowing of background activity, reflecting cortical dysfunction secondary to reduced cerebral perfusion; (b) decreased alpha/delta power ratio (ADR), which quantifies the shift from normal alpha-dominant activity to pathological slow-wave predominance—Balança et al. demonstrated that a >30% decrease in ADR achieved 100% sensitivity and 89% specificity for predicting DCI 6–24 hours before clinical onset [25]; (c) loss of normal sleep architecture and EEG variability, which may reflect early cortical metabolic compromise; and (d) epileptiform activity, including periodic discharges and electrographic seizures, which can indicate cortical irritability from ischemia and have been associated with worse outcomes.

Quantitative EEG (qEEG) analysis using automated algorithms has shown particular promise. Zheng et al. (2022) demonstrated that machine learning–based EEG analysis could detect ischemic patterns by identifying changes in relative alpha variability and coherence that precede clinical deterioration [26]. Chen et al. further showed that combining TCD hemodynamic data with EEG features provided superior DCI prediction (sensitivity 90%, specificity 70%) compared to either modality alone [27], suggesting that multimodal integration enhances detection. Studies using automated qEEG analysis (predominantly post-2018) demonstrated higher accuracy than earlier studies relying on visual interpretation alone, likely reflecting advances in signal processing and pattern recognition algorithms. A key advantage of cEEG is its capacity for continuous, real-time monitoring at the bedside, enabling detection of evolving ischemia even in sedated or poor-grade patients who cannot be reliably examined clinically. However, implementation requires dedicated neurophysiology expertise and infrastructure for continuous monitoring and interpretation.

### Emerging Technologies

Near-Infrared Spectroscopy (NIRS): Park et al. (2020) established specific NIRS threshold (>12.7% rSO2 reduction) with 94.44% sensitivity for DCI detection in a single small cohort (n=52). This promising finding requires validation in larger, multicenter studies before clinical adoption [10].

Cerebral Microdialysis: Comprehensive review by Helbok et al. demonstrated microdialysis provides earliest markers of ischemia (lactate/pyruvate ratio >40, glucose <0.7 mmol/L) [11].

### Predictors of Delayed Cerebral Ischemia

We identified 84 studies investigating DCI predictors, including 23 ML-based models.

### Clinical Predictors

Poor admission neurological grade emerged as the strongest predictor, with Hunt-Hess grade IV-V carrying a pooled odds ratio of 3.87 (95% CI: 3.21-4.66) for DCI [29]. Notably, a GCS-based grading system has demonstrated greater interrater reliability (κ=0.46) compared to Hunt-Hess (κ=0.41) and WFNS (κ=0.27) in validation studies, and may provide superior prognostic discrimination due to its broader familiarity among medical personnel and avoidance of ambiguous terms like "stupor" [29, 30]. The 2023 AHA/ASA guidelines support use of either Hunt-Hess or WFNS grading, though clinical practice increasingly favors GCS-based assessment.

### Radiological Predictors

The Modified Fisher scale (not the original Fisher scale) demonstrated the strongest association with DCI among radiological markers. It is important to note the distinction between the original Fisher scale and the Modified Fisher scale. In the original Fisher scale (1980), Grade 4 (diffuse/thin SAH with IVH/ICH) paradoxically had LOWER vasospasm risk than Grade 3. The Modified Fisher scale (Frontera et al., 2006) was developed specifically to address this counterintuitive feature, with Grade 4 now representing the highest risk category [31]. Throughout this review, we use the Modified Fisher scale unless otherwise specified.

### Laboratory Biomarkers

The field of biomarker research has experienced substantial growth, with several markers showing promising results [32–35].

### Machine Learning Models

A 2025 systematic review by Zhang et al. (48 studies, 16,294 patients, 71 ML models) provides the most comprehensive assessment [36]. While ML models incorporating multimodal data showed improved prediction compared to traditional risk factors (AUC 0.68-0.88 vs. ~0.63), it is important to note that only 31 of 71 models (44%) underwent independent validation, and 67% of studies demonstrated high risk of bias per PROBAST assessment [36]. These findings suggest promising but preliminary evidence requiring further prospective validation before clinical implementation [9, 37–40].

## Quality Assessment

Application of the QUADAS-2 tool [13] to diagnostic accuracy studies revealed generally high methodological quality in key domains. Patient selection showed low risk of bias in 88% of studies. Index test conduct demonstrated low risk of bias in 92% of studies. However, a significant limitation emerged in the flow and timing domain, where 35% of studies showed high risk of bias due to variable intervals between index and reference tests.

Prediction model studies assessed using the PROBAST tool [14] revealed more concerning quality issues, with 67% demonstrating high risk of bias. These quality limitations should inform interpretation of reported diagnostic performance and predictive accuracy.

## Economic Considerations

Thompson et al. (2022) [43] comprehensive review documented substantial cost variation across surveillance modalities.

## Discussion

### Principal Findings

In this scoping review spanning 2005–2025, we found convergent evidence that no single modality provides sufficient sensitivity, specificity, and practicality to serve as a standalone solution for vasospasm surveillance. Rather than providing prescriptive recommendations, the evidence suggests that multimodal strategies anchored in contemporary guideline recommendations may offer the most robust path to timely detection [5].

Among noninvasive methods for DCI detection, CT perfusion (CTP) consistently demonstrated high diagnostic accuracy, with pooled sensitivities and specificities approaching 80–86%. However, it is essential to recognize that CTP measures hypoperfusion—a consequence of impaired cerebral blood flow—rather than vasospasm itself. CTP cannot determine whether perfusion meets metabolic demand; PET with oxygen extraction fraction remains the gold standard for metabolic assessment [21, 22].

As detailed in the Results, DSA was used as the reference standard for all vasospasm detection studies and was not evaluated as a separate detection modality. Its unique role encompasses both definitive diagnosis of angiographic vasospasm and the ability to provide immediate endovascular treatment (intra-arterial vasodilators, angioplasty) when indicated. This distinction is clinically important: DSA identifies vasospasm (arterial narrowing), whereas DCI—as defined by the 2010 consensus criteria—is a clinical and radiographic diagnosis that can occur through multiple mechanisms beyond vasospasm, including microthrombosis, cortical spreading depolarization, and neuroinflammation.

### Temporal trends in diagnostic accuracy

A notable observation from our review is the improvement in diagnostic accuracy over time, particularly for technology-dependent modalities. CTP studies published after 2015 demonstrated approximately 10% higher sensitivity and specificity compared to earlier studies, likely reflecting advances in scanner technology (faster acquisition, wider coverage), post-processing algorithms, and protocol standardization. Similarly, quantitative EEG analysis using machine learning approaches (predominantly post-2018) outperformed earlier visual interpretation methods. While formal meta-regression was beyond the scope of this scoping review, these temporal trends suggest that older studies may underestimate current diagnostic performance and highlight the need for updated systematic reviews with meta-regression as an important avenue for future research.

### Multimodal Neuromonitoring

**Fig. 2:**
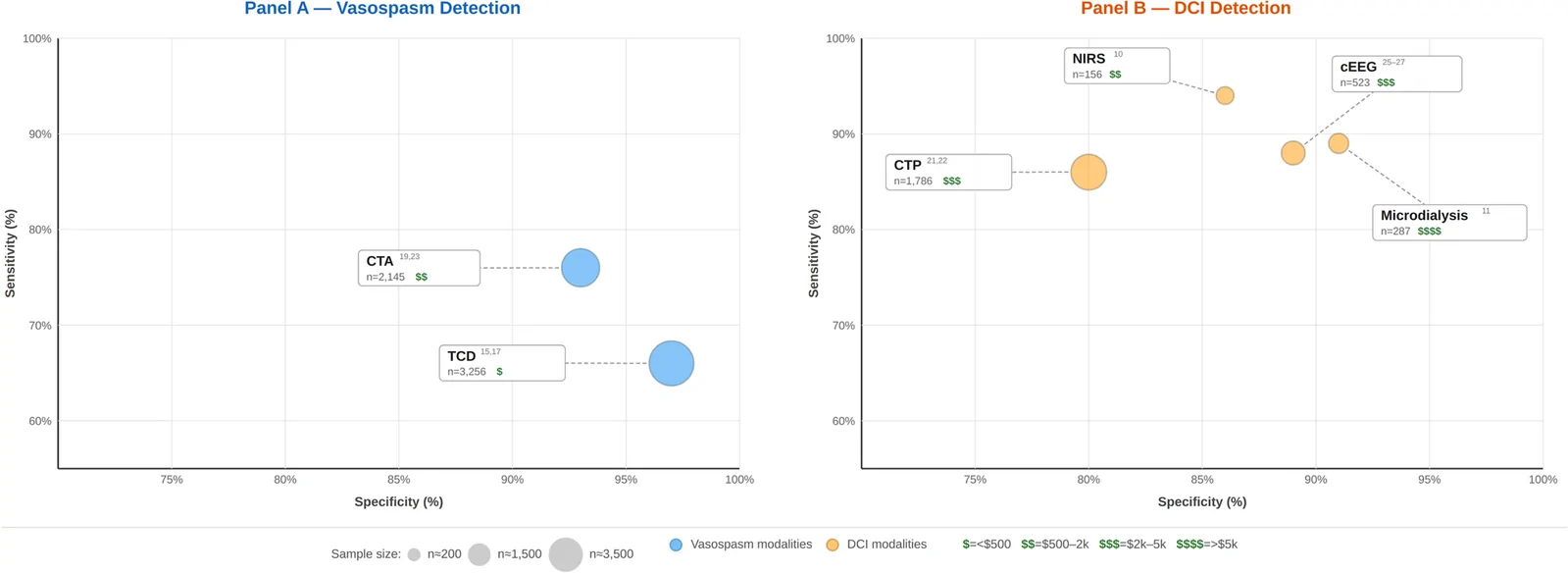
Diagnostic performance of vasospasm and DCI surveillance modalities. Legend. Two-panel scatter plot displaying comparative diagnostic performance of surveillance modalities after aneurysmal subarachnoid hemorrhage. Each modality is plotted according to its pooled sensitivity (y-axis, range 60–100%) and specificity (x-axis, range 85–100%) based on meta-analyses and systematic reviews. The size of each data point corresponds to the number of patients evaluated (larger circles indicate more patients). Relative cost per examination is indicated by the number of dollar signs ($). Panel A (Vasospasm Detection, DSA as reference standard) shows TCD and CTA performance. Panel B (DCI Detection, 2010 consensus criteria) shows CTP, cEEG, NIRS, and microdialysis performance. DSA is not plotted as it serves as the reference standard, not a detection modality. TCD = Transcranial Doppler; CTA = Computed Tomography Angiography; CTP = Computed Tomography Perfusion; DSA = Digital Subtraction Angiography; cEEG = continuous Electroencephalography; NIRS = Near-Infrared Spectroscopy

The evidence base for continuous multimodal neuromonitoring in SAH is growing [41] but remains limited by small sample sizes, single-center designs, and heterogeneous protocols. The TITAN study (Gouvêa Bogossian et al., 2025; 232 patients, 5 centers) examined the association between brain tissue hypoxia (low PbtO2) and metabolic dysfunction (elevated lactate/pyruvate ratio) with 6-month neurological outcomes (Glasgow Outcome Scale). Importantly, this study assessed physiological associations with long-term functional outcomes rather than DCI detection or prediction per the 2010 consensus criteria; therefore, its findings should not be extrapolated to DCI-specific surveillance protocols [28]. Evidence that monitoring-guided therapy improves patient outcomes remains limited, and multimodal monitoring should be considered primarily at experienced high-volume centers where expertise and infrastructure exist (Fig. 2).

### Clinical implications

**Fig. 3:**
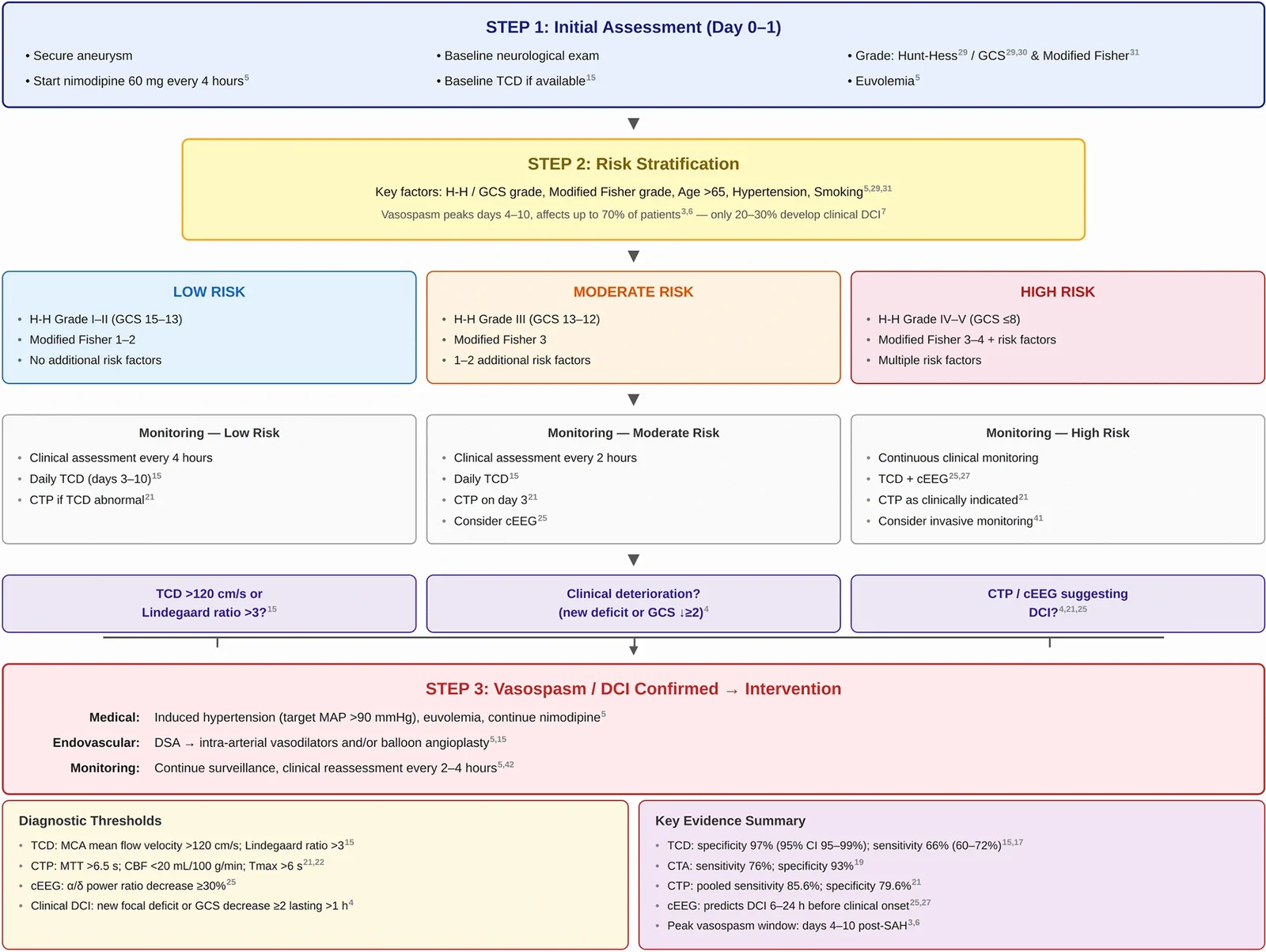
Clinical decision algorithm for vasospasm & DCI surveillance after SAH. Legend. H–H = Hunt-Hess; GCS = Glasgow Coma Scale (GCS ≤ 8 = poor grade); RF = risk factors; TCD = transcranial Doppler; CTP = CT perfusion (measures hypoperfusion); CTA = CT angiography; DSA = digital subtraction angiography; cEEG = continuous electroencephalography. Note: This algorithm represents a synthesis of available evidence and expert consensus rather than a validated protocol. Individual components vary in their level of validation and should be applied with clinical judgment. Modified Fisher scale is used throughout (Grade 4 = thick cisternal blood WITH IVH = highest risk)

Taken together, these data support a tiered, risk-adapted surveillance paradigm aligned with guideline statements [5, 42]. A proposed clinical decision framework is presented in Figure 3. This framework represents a synthesis of available evidence and expert consensus rather than a validated protocol, and individual components should be applied with clinical judgment based on institutional capabilities and patient characteristics. For clinical grading, while Hunt-Hess and WFNS scales remain widely used, clinicians should be aware that GCS-based assessment may offer superior interrater reliability and prognostic accuracy. Figure 3 incorporates GCS thresholds (e.g., GCS ≤8 for poor grade) alongside traditional grading scales.

### Implementation barriers and solutions

The principal barriers to widespread adoption of advanced surveillance technologies include cost, expertise requirements, and lack of standardization [44].

### Emerging technologies and future directions

**Fig. 4:**
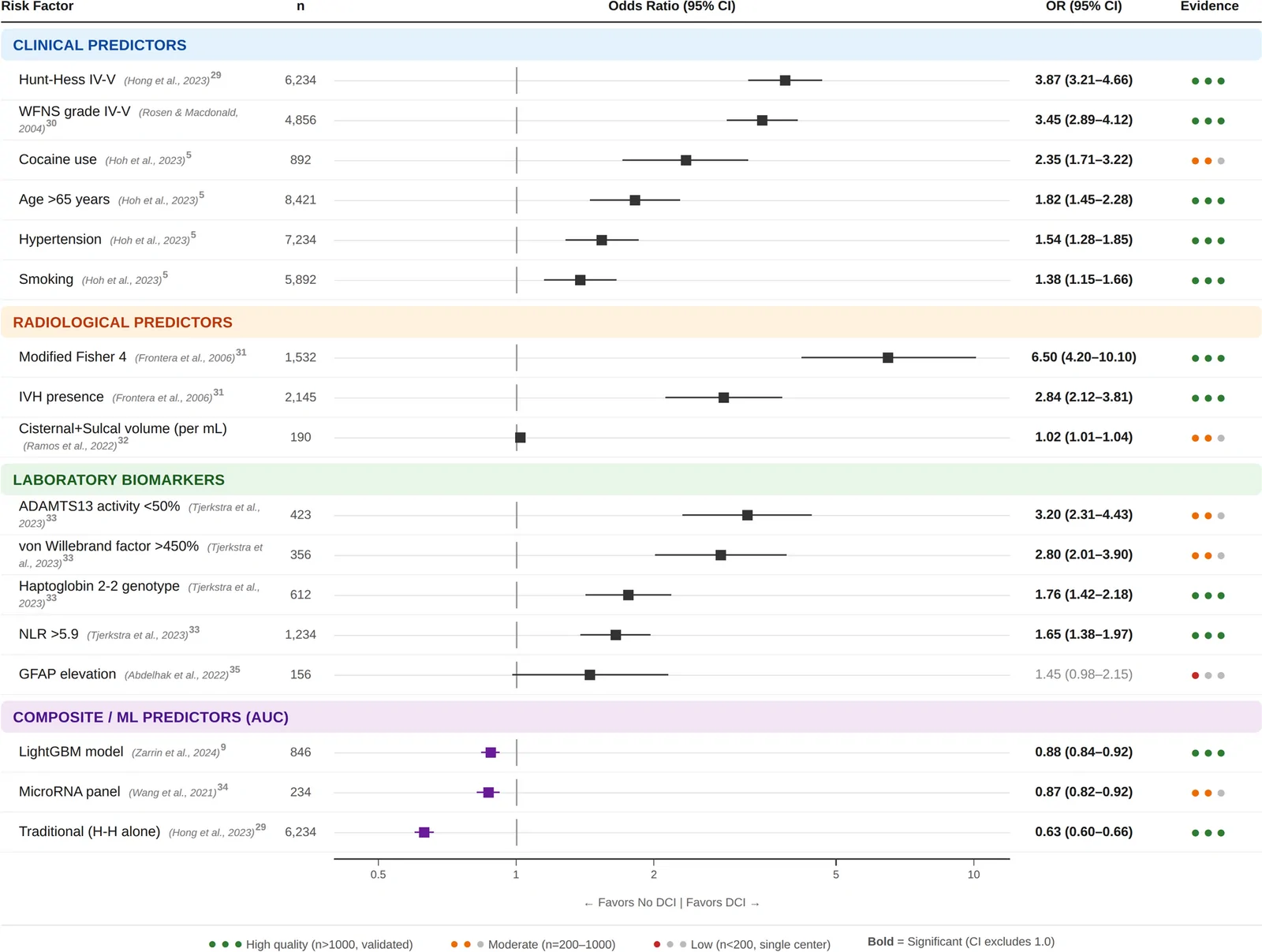
Risk factors for delayed cerebral ischemia after aneurysmal SAH. Legend. OR = odds ratio; CI = confidence interval; AUC = area under curve; H–H = Hunt-Hess; WFNS = World Federation of Neurological Surgeons; IVH = intraventricular hemorrhage; NLR = neutrophil-to-lymphocyte ratio; GFAP = glial fibrillary acidic protein; ML = machine learning; DCI = delayed cerebral ischemia

Several emerging technologies merit particular emphasis given their growth trajectory (Fig. 4).

### Limitations

Some limitations should be acknowledged. First, heterogeneity in outcome definitions remains a significant barrier; despite the 2010 consensus criteria, many studies continue to use "symptomatic vasospasm" or other non-standardized definitions, limiting comparability. Second, the scoping review methodology precluded formal meta-analysis or meta-regression examining the effect of publication year on diagnostic accuracy—an important avenue for future systematic reviews given technological advances over the 20-year study period. We partially addressed this through descriptive analysis by publication era, which revealed improving diagnostic performance over time, but formal meta-regression remains warranted.

Modality-specific limitations include: TCD operator dependence and inability to assess posterior/distal circulation; CTA radiation exposure and limited sensitivity for distal vasospasm; CTP's measurement of hypoperfusion rather than vasospasm or metabolic adequacy; and the invasiveness and resource requirements of microdialysis and PbtO2 monitoring. For prediction models, high risk of bias (67% per PROBAST) and limited external validation (only 44% of ML models) temper interpretation of reported accuracy metrics.

## Conclusions

Optimal surveillance for cerebral vasospasm after SAH requires multimodal approaches tailored to individual risk profiles, as emphasized in the 2023 AHA/ASA guidelines. Clear distinction between vasospasm (angiographic narrowing, DSA gold standard) and DCI (2010 consensus definition) is essential for appropriate test selection and interpretation. The clinical decision algorithm presented represents a framework based on available evidence rather than a validated protocol, and should be applied with clinical judgment. Multimodal monitoring shows promise but evidence for outcome improvement remains limited. Machine learning approaches demonstrate encouraging predictive performance but require prospective validation before clinical implementation.

## Supplementary Information

Below is the link to the electronic supplementary material.

Supplementary file1 (TIFF 1113 KB)
